# Leisure-Time Physical Activity, Sleep, and Self-Perception of Health During the COVID-19 Lockdown in Québec

**DOI:** 10.3390/epidemiologia7040117

**Published:** 2026-08-20

**Authors:** Adrien Murphy-Després, Natalie Alméras, Dominic J. Chartrand, Isabelle Lemieux, Serge Simard, Laura Jalbert, Annie LeBlanc, Jean-Pierre Després

**Affiliations:** 1 Centre de recherche de l’institut universitaire de cardiologie et de pneumologie de Québec – Université Laval, Québec, QC G1V 4G5, Canada; adrien.murphy-despres.1@ulaval.ca (A.M.-D.); natalie.almeras@criucpq.ulaval.ca (N.A.); dominic.chartrand@criucpq.ulaval.ca (D.J.C.); isabelle.lemieux@criucpq.ulaval.ca (I.L.); serge.simard@criucpq.ulaval.ca (S.S.); 2 Department of Kinesiology, Faculty of Medicine, Université Laval, Québec, QC G1V 0A6, Canada; 3 VITAM – Centre de recherche en santé durable, Québec, QC G1J 2G1, Canada; laura.jalbert.ciussscn@ssss.gouv.qc.ca (L.J.); annie.leblanc@fmed.ulaval.ca (A.L.)

**Keywords:** COVID-19 lockdown, mental health, physical activity, physical health, sleep quality

## Abstract

*Background and Objectives*: The lockdown imposed by public health authorities during the COVID-19 pandemic has disrupted many behaviours, including physical activity (PA). This study aimed to assess changes in selected components of the 24 h movement behaviours during the COVID-19 lockdown and to examine whether changes in these components were associated with changes in self-perceived physical health, mental health and sleep quality. *Materials and Methods*: The sample was drawn from the MAVIPAN study, a mixed-methods based, prospective, observational, longitudinal cohort study. In the present analyses, we used cross-sectional self-reported data collected through online questionnaires. Participants in the present analyses had to be 18 years of age and have complete gender and PA data (*n* = 2268). *Results*: During the lockdown, reductions in total PA (effect size: −8.4%; 95% CI: −11.9%, −4.7%; *p* < 0.0001) and moderate-to-vigorous-intensity PA (MVPA) (effect size: −8.7%; 95% CI: −13.1%, −4.1%; *p* < 0.0001) were observed. Total PA increased during the lockdown among participants who self-perceived their physical health, mental health, and sleep quality as better compared to those who reported worse outcomes (*p* < 0.0001). After adjusting for several confounding variables, an increase in MVPA of 30 min per week was associated with higher odds of reporting better physical health (1.65; 95% CI: 1.51, 1.81; *p* < 0.0001). *Conclusions*: Although our study design does not allow causal inference, these findings are concordant with public health strategies considering PA to help enhance and maintain physical and mental health, including sleep quality, in both constrained and everyday conditions.

## 1. Introduction

On 31 December 2019, the first severe acute respiratory syndrome coronavirus-2 (SARS-CoV-2) cases were reported in Wuhan, China [1]. Three months later, the World Health Organization declared a global pandemic [1]. In the province of Québec in Canada, the government declared a “Sanitary State of Emergency” on 13 March 2020, forcing all employees to work remotely and all nonessential businesses to temporarily close down [2]. All gatherings were prohibited and going outside was only allowed if the activity involved family members/roommates residing at the same address. Furthermore, travelling across different regions was restricted by local authorities. Schools, from elementary to university, switched to online classes, disrupting the lives of parents and students who needed to adapt to this unprecedented crisis. Quebecers were asked to practice social distancing, which required always keeping a 2 m distance between individuals from different households. As a result of the lockdown, all physical activity (PA) centres, including (but not limited to) fitness centres, ice rinks, pools, and even outdoor parks, were closed to reduce the spread of the virus. Outdoor PA was allowed within a 1 km distance from the personal residence.

As a consequence, practicing regular PA became more complicated, and many international studies reported a global decrease in PA levels during the COVID-19 pandemic. For instance, PA was reduced and sedentary time was increased in youth, adult, or elderly populations during the lockdowns [3,4], resulting in fewer people reaching weekly PA recommendations [5,6]. However, some studies have reported that the largest decrease in PA was observed in previously active people, while previously inactive people did not change or even slightly increased their PA practice [5,7,8,9]. Other studies have reported mixed results, although an overall trend towards a decrease in PA was observed [10], suggesting a heterogeneous response to lockdown measures regarding PA practice. Given the known associations between PA, sedentary behaviour and mental health, especially anxiety and depressive symptoms [11,12,13], it was of no surprise to note that studies conducted during the lockdowns had shown an association between a decrease in PA and a decrease in well-being and mental health [3,8,9]. The early phase of the pandemic has also dramatically affected sleep patterns [14]. Studies reported that sleep quality, either self-reported or measured by online questionnaires, was globally reduced during lockdowns [15,16,17], especially among previously active people [9]. However, it is interesting to note that in a subsample of people who reported insomnia before the pandemic, sleep quality was increased during lockdown, suggesting once again differences in individual responses [16].

In Canada, it was reported that PA remained relatively stable in the adult population and that PA was linked to a better perception of mental and general health while screen time was linked to a worsened health perception [18,19]. However, to the best of our knowledge, very limited quantitative data is available to understand how behaviours and related dimensions of health changed during the lockdown in the province of Québec.

Therefore, the purpose of the present analyses was to assess changes in selected components of 24 h movement behaviours among adults living in Québec in the context of the COVID-19 lockdown, and to document the associations between changes in leisure-time PA and self-perceived physical health, mental health, and sleep quality. We tested the hypothesis that individuals who increased their PA level during the lockdown would report more favourable changes in physical and mental health, including sleep quality.

## 2. Materials and Methods

### 2.1. Study Design

The MAVIPAN (Ma vie et la pandémie/My Life and the Pandemic) study is a mixed-methods based, prospective, observational, longitudinal cohort study, which was launched on 29 April 2020 in the province of Québec, Canada (ClinicalTrials.gov Identifier: NCT04575571) [20]. Using quantitative and qualitative data reported in online surveys, the two main objectives of the present study were to describe changes in behaviours and related health dimensions in the population during the pandemic (cross-sectional component) and to examine how participants adapted to the crisis over time (longitudinal aspect) [20]. The complete protocol has already been published elsewhere [20]. The MAVIPAN study was approved by the Research Ethics Board of the Primary Care and Population Health Research Sector of the Centre intégré universitaire de santé et de services sociaux de la Capitale-Nationale (CIUSSS de la Capitale-Nationale) (#2021–2043).

### 2.2. Participants

Participants over 14 years of age who understood French or English and lived in the province of Québec were recruited using non-probabilistic sampling, through the study website, local media, social media, as well as by mass diffusion in healthcare centres and universities [20]. Every participant gave his/her informed consent before taking the online MAVIPAN survey. As of 17 July 2020, 2663 participants had completed the first part of the study. To be included in the present analyses, participants had to be 18 years of age (51 participants were excluded because their age was <18 years), had to report their gender (7 participants were excluded because they were gender-fluid, non-binary and/or Two-Spirit or did not answer) as well as provide valid PA data (337 participants excluded: 266 participants had missing PA data and 71 had discordant PA data) before and during the lockdown (Supplementary Figure S1). The final sample included 2268 participants.

### 2.3. Measures

Sociodemographic variables and self-reported general health, chronic disease diagnoses, and PA data were collected. At the same time point in the survey, participants had to recall their behaviours (PA data and self-perceived changes in physical health, mental health, and sleep quality). Therefore, data before the lockdown were retrospectively reported. Most of the MAVIPAN questionnaires included validated scales; however, items assessing PA, screen time, sleep duration, and sleep quality were not validated. Items assessing self-perceived physical and mental health were derived from a validated questionnaire. Additional information on questionnaires used in the MAVIPAN study is available in the protocol publication [20]. It is also relevant to mention that, as MAVIPAN was open to anyone living in the province of Québec, each administrative region had different confinement measures as the spread of the virus was faster in certain regions, especially around Montréal and Québec City. As a result, some people, despite living in the same province, may have experienced completely different lockdown conditions depending on where they lived at the time of the survey completion.

#### 2.3.1. Anthropometric Measurements

Participants were asked to report their weight and height to calculate the body mass index (BMI) in kg/m^2^.

#### 2.3.2. Selected Components of the 24 h Movement Behaviours

Leisure-time PA (time spent doing activities that increase heart rate) was reported in minutes/week. Moderate-to-vigorous-intensity PA (MVPA) was estimated from activities perceived as making participants somewhat out of breath, a self-perceived indicator of exercise intensity based on the same physiological principle as the Talk Test [21] and commonly used to distinguish MVPA in population-based surveys [22]. Data were considered discordant if participants reported doing more MVPA than total PA, and they were then removed from the dataset. Light-intensity PA (LPA) was calculated by subtracting MVPA from total leisure-time PA. Participants were categorized into three subgroups: “Increased”, “Unchanged” and “Reduced”. Recreational screen time (outside of work), which was used as an estimate of sedentary behaviour, was reported in minutes/day. Sleep time was reported in hours/day.

#### 2.3.3. Self-Perceived Physical and Mental Health

Self-perceived changes in physical and mental health during the lockdown compared to before were reported with five different options: “Much better now”, “A little better now”, “About the same”, “A little worse now” or “A lot worse now”. The two negative answers and the two positive answers were both grouped, resulting in three different subgroups: “Better”, “Unchanged” and “Worse”.

#### 2.3.4. Self-Perceived Sleep Quality

Participants were asked if their sleep quality was affected since the beginning of the lockdown. Four options were made available: “No, it’s better now”, “No, about the same”, “Yes, a little worse” or “Yes, much worse”. The two negative answers were grouped, and therefore results are presented according to three subgroups: “Better”, “Unchanged” and “Worse”.

### 2.4. Statistical Analyses

Sociodemographic variables are reported as the number of participants and the percentage of the sample in parentheses. Quantitative measures such as anthropometric measurements, total leisure-time PA, MVPA, LPA, and sleep time are reported as means ± 95% confidence intervals (CI) in tables. Data in figures are presented as proportions or as means ± standard error.

**Figure 1:**
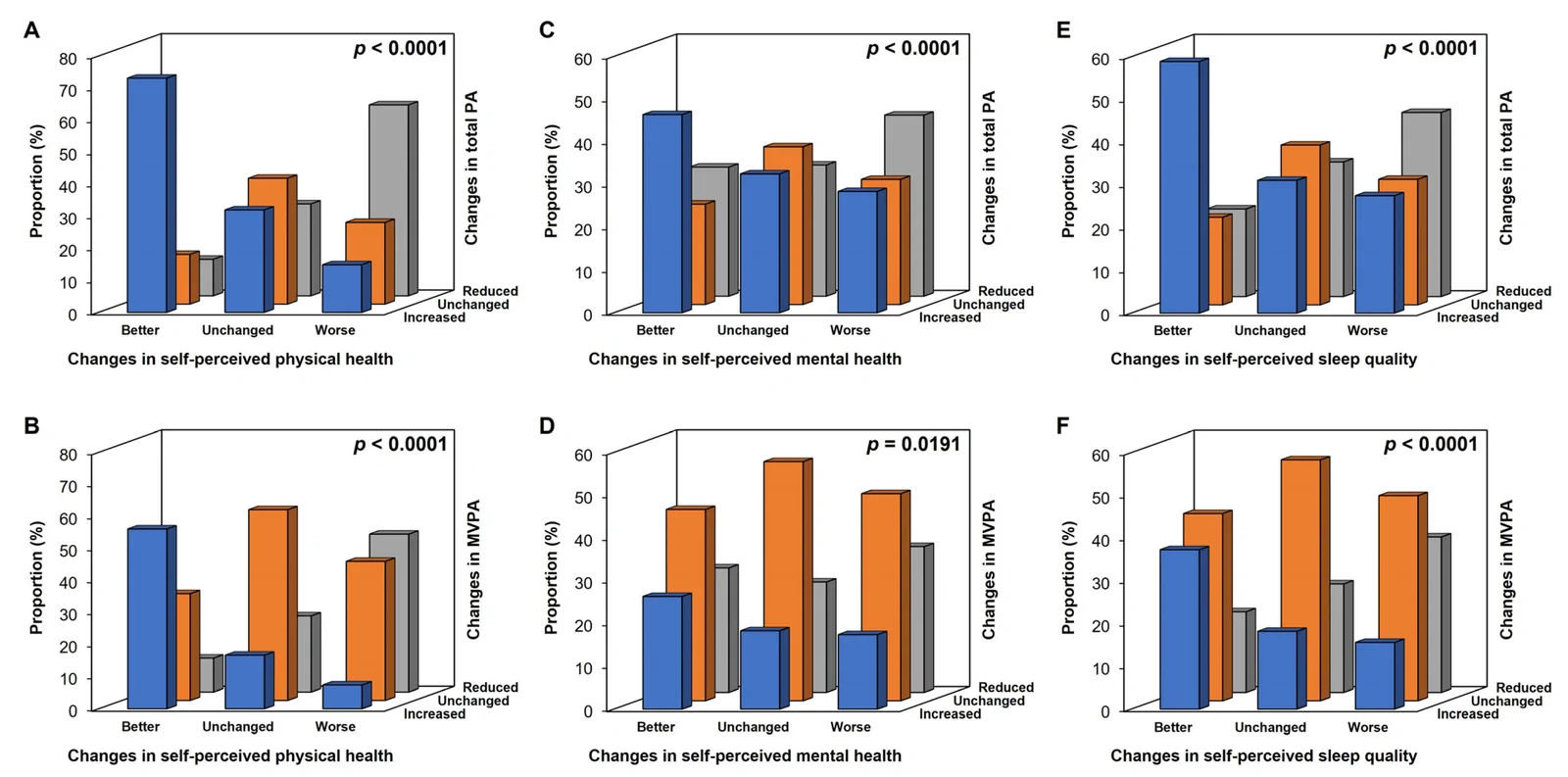
Changes in self-perceived physical health (**A**,**B**), mental health (**C**,**D**), and sleep quality (**E**,**F**) according to changes in total leisure-time physical activity (PA) or moderate-to-vigorous-intensity PA (MVPA).

**Figure 2:**
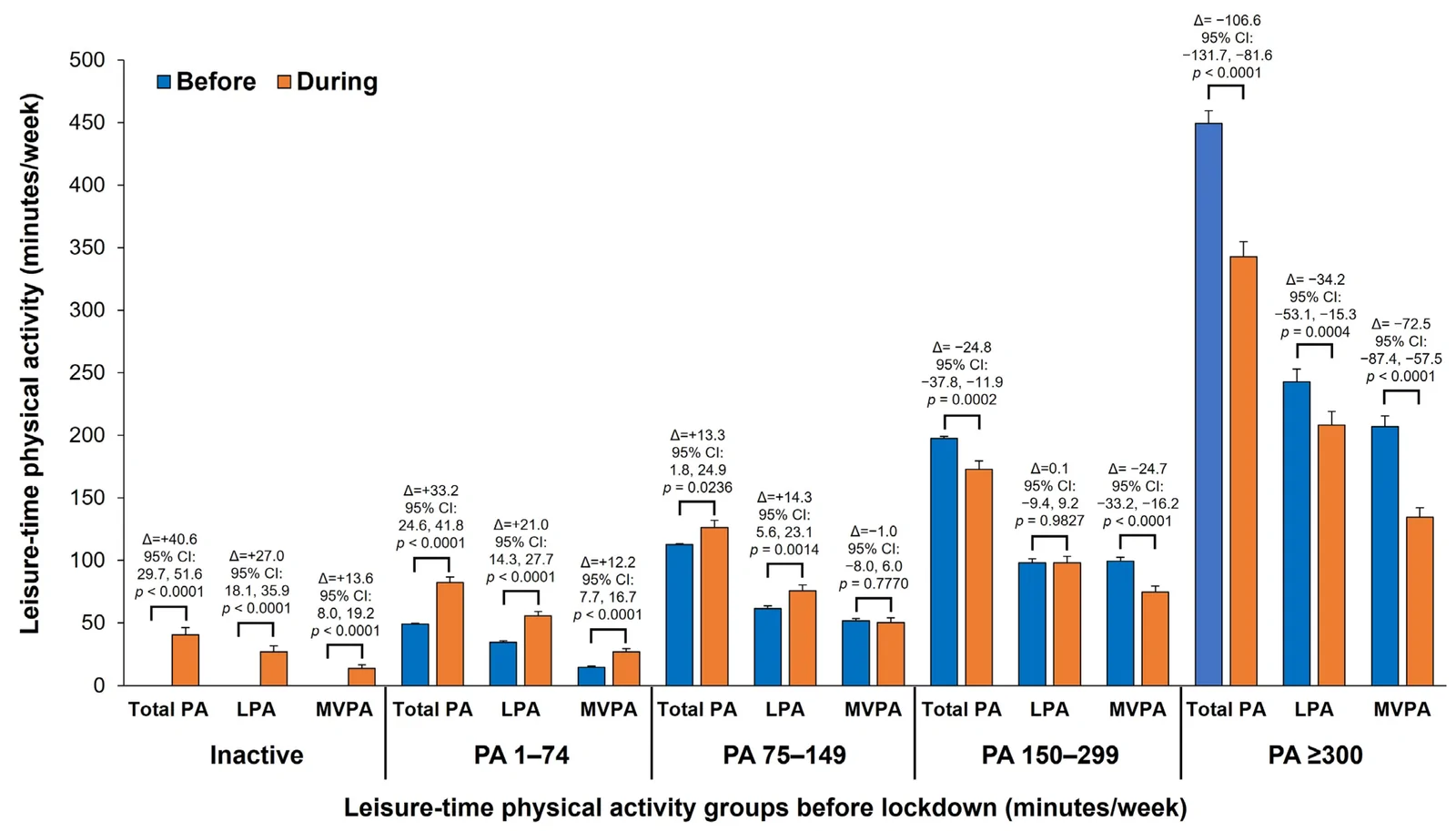
Total leisure-time physical activity (PA), light-intensity PA (LPA), and moderate-to-vigorous-intensity PA (MVPA) before and during the lockdown according to total leisure-time PA in minutes/week before the lockdown.

Comparisons between better and worse groups of changes in self-perceived physical health, mental health and sleep quality were analyzed using the Chi-square statistic (Figure 1). Paired *t*-tests were used to compare PA variables before and during the lockdown according to PA levels before the lockdown (Figure 2). Unpaired *t*-tests were used to compare data between women and men (Supplementary Figures S2–S4).

Recreational screen time, sleep time and the percentage of participants meeting the 150 min/week of MVPA were analyzed using generalized linear mixed models, using a lognormal distribution for screen and sleep time, and a binary distribution for the percentage of participants meeting the MVPA guidelines. To compare behaviours before and during lockdown, a fixed factor related to the time effect (during vs. before lockdown) and a random factor related to the subjects were defined (Table 2 and Results section). To compare changes in self-perceived physical health, mental health and sleep quality (Tables 3–5) or healthcare workers and non-healthcare workers (Supplementary Table S1 and Results section), a second fixed factor was added to the statistical model (group effect) with an interaction term between these two fixed factors.

Leisure-time PA variables (total PA, LPA and MVPA) were analyzed using a two-part mixture model combining a zero-inflated binomial component and a normal component, both accounting for repeated measures (Tables 2–5 and Table S1). These variables were characterized by the presence of a large proportion of zero values, in addition to continuous positive values. The probability density of the data had a mass at 0 with probability *p*, and a normal density function was defined on the positive values with probability 1 − *p*. For both parts of the model, a fixed factor associated with the comparison between before and during lockdown (time effect) was defined to explain the process that generates zeros, as well as another fixed factor to explain the distribution of positive values. When necessary, a second fixed factor, different for both parts of the model, was defined for comparisons among groups with an interaction term between the two fixed factors. The initial parameter estimate values for the zero-inflated part of the nonlinear mixed model were derived from a generalized linear mixed model defining variables as a binary variable. A linear mixed model on values > 0 was performed to get initial values for the normal distribution part of the nonlinear mixed model. The dependence among residuals of repeated measurements within the same experimental unit was estimated with an unstructured covariance association. The normality hypothesis was verified using the Shapiro–Wilk test on residuals from the statistical model on positive values and transformed by the Cholesky metric. Brown and Forsythe’s variation of Levene’s test statistic was used to verify the homogeneity of variances. To fulfil these assumptions, the selected components of the 24 h movement behaviours were log-transformed. The effect size reported in Table 2 was expressed as the percentage change between the geometric means of the compared conditions, obtained by back-transforming the estimated difference on the log scale: Effect Size (%) = exp(Estimate) − 1. For the zero-inflated values, effect size was expressed as a risk difference, defined as the difference between the estimated proportions (marginal means, back-transformed via the inverse link function) for the two levels of the “time effect”. The standard error of the risk difference was obtained using the delta method, based on the variance-covariance matrix of the logit-scale estimates, considering the correlation between the two estimates. Ninety-five percent CIs were calculated using the asymptotic normal approximation.

Univariable and multivariable logistic regressions were performed to determine factors independently associated with better physical health, mental health and sleep quality according to an increase in selected components of the 24 h movement behaviours (Table 6). Continuous variables were checked for the assumption of linearity in the logit using graphical representations. For the multivariable logistic regression analyses, results were adjusted for several confounding variables such as age, BMI, presence of reported diabetes, hypertension, hypercholesterolemia, and heart disease, baseline total PA, education, household income, working status, and administrative regions. Statistical significance was set at ≤0.05. All analyses were performed with SAS v9.4 (SAS Institute, Cary, NC, USA).

## 3. Results

After applying the exclusion criteria, the present analyses were performed on a cohort of 2268 participants (see Supplementary Figure S1 for information on the number and reasons for participants’ exclusion). The 337 participants excluded on the basis of PA data had similar characteristics (age, gender, BMI, baseline total PA, MVPA, and LPA levels) as the 2268 participants, except for education. Participants included in these analyses answered the survey after a lockdown of 2 to 5 months, depending on the completion date. Table 1 presents sociodemographic data of the cohort. Participants were mostly women (82%) with a mean age of 44 ± 14 years. Most participants were Caucasian (80%), a large proportion had obtained at least a university undergraduate degree (71%), and 30% had a household income of 100,000 CAD or more. About 75% of participants were from two administrative regions (Capitale-Nationale and Chaudière-Appalaches), but all administrative regions of the province were represented. Mean BMI was 26.3 ± 5.6 kg/m^2^ and the prevalence of reported diabetes, hypertension, hypercholesterolemia, and heart disease was 3.4%, 9.5%, 3.7%, and 1.9%, respectively.

**Table 1:** Participants’ sociodemographic information.

Variables	*n* (% of Sample)
Gender (*n* = 2268)	
Women	1868 (82.4)
Men	400 (17.6)
Working status (*n* = 2265)	
Working before the lockdown	1739 (76.8)
Not working before the lockdown	524 (23.1)
Do not wish to answer	2 (0.1)
Legal status (*n* = 2240)	
Canadian citizen	2192 (97.9)
Permanent resident	20 (0.9)
Temporary resident	26 (1.2)
Others	2 (0.1)
Civil status (*n* = 2242)	
Single	440 (19.6)
Married	618 (27.6)
Common-law couple	953 (42.5)
Divorced or separated	189 (8.4)
Widower	36 (1.6)
Do not wish to answer	6 (0.3)
Ethnic ancestry (*n* = 2003)	
European	918 (45.8)
Other North American origins	688 (34.3)
North American Aboriginal origins	78 (3.9)
African	17 (0.9)
Asian	15 (0.8)
Latin American, South/Central America	18 (0.9)
Caribbean	6 (0.3)
Other	82 (4.1)
Do not wish to answer	181 (9.0)
Education (*n* = 2266)	
High school not completed	17 (0.8)
High school or equivalent	198 (8.7)
College	443 (19.5)
Undergraduate	821 (36.2)
Graduate	782 (34.5)
Do not wish to answer	5 (0.2)
Household income, CAD (*n* = 2238)	
<10,000	21 (0.9)
10,000–19,999	72 (3.2)
20,000–29,999	78 (3.5)
30,000–39,999	72 (3.2)
40,000–49,999	114 (5.1)
50,000–59,999	133 (5.9)
60,000–69,999	102 (4.6)
70,000–79,999	124 (5.5)
80,000–89,999	123 (5.5)
90,000–99,999	148 (6.6)
≥100,000	679 (30.3)
Do not know	32 (1.4)
Do not wish to answer	540 (24.1)

Before the lockdown, only 19.4% (95% CI: 17.6, 21.3) of the sample reached the latest Canadian guidelines of 150 min/week of MVPA [23] and this number dropped to 16.0% (95% CI: 14.4, 17.8) during the lockdown (*p* = 0.0004). Selected components of the 24 h movement behaviours before and during the lockdown are shown in Table 2. According to the zero-inflated portion of the model, the proportion of individuals not doing PA during the lockdown increased (effect size: 4.3%; 95% CI: 2.6%, 5.9%; *p* < 0.0001) which yielded a decrease in total leisure-time PA duration (effect size: −8.4%; 95% CI: −11.9%, −4.7%; *p* < 0.0001). These results were mainly attributable to changes observed in MVPA (effect size: 10.2%; 95% CI: 8.3%, 12.1%; *p* < 0.0001 and effect size: −8.7%; 95% CI: −13.1%, −4.1%; *p* < 0.0001 for the zero-inflated portion of the model and duration, respectively), while no significant changes were observed in LPA. An increase in recreational screen time (effect size: 43.2%; 95% CI: 40.5%, 46.0%; *p* < 0.0001) and a slight decrease in sleep duration were also observed during the lockdown (effect size: −2.8%; 95 CI: −3.5%, −2.1%; *p* < 0.0001). Finally, the proportion of participants simultaneously meeting guidelines for MVPA, recreational screen time, and sleep duration decreased from 10.4% to 5.1% during the lockdown.

**Table 2:** Selected components of the 24 h movement behaviours before and during lockdown.

Variables		Before Mean (95% CI)	During Mean (95% CI)	Effect Size (95% CI)	*p* Value Time
Total PA *, min/week	Zero-inflated	17.7% (16.2%, 19.4%)	22.0% (20.3%, 23.8%)	4.3% (2.6%, 5.9%)	<0.0001
		130.5 (124.8, 136.5)	119.6 (114.2, 125.3)	−8.4% (−11.9%, −4.7%)	<0.0001
LPA *, min/week	Zero-inflated	35.0% (33.1%, 37.0%)	36.1% (34.1%, 38.1%)	1.1% (−0.8%, 2.9%)	0.2898
		79.4 (74.7, 84.5)	80.5 (75.6, 85.7)	1.3% (−2.9%, 5.7%)	0.6890
MVPA *, min/week	Zero-inflated	42.9% (40.8%, 44.9%)	53.0% (51.0%, 55.1%)	10.2% (8.3%, 12.1%)	<0.0001
		78.1 (72.2, 84.5)	71.3 (65.5, 77.7)	−8.7% (−13.1%, −4.1%)	<0.0001
Screen time ^†^, min/day		140.0 (136.4, 143.6)	200.5 (195.3, 205.8)	43.2% (40.5%, 46.0%)	<0.0001
Sleep, hours/day		7.5 (7.4, 7.5)	7.3 (7.2, 7.3)	−2.8% (−3.5%, −2.1%)	<0.0001

CI, confidence interval; LPA, light-intensity physical activity; MVPA, moderate-to-vigorous-intensity physical activity; PA, physical activity. * Physical activity performed during leisure time; ^†^ Recreational screen time. The effect size is expressed as a percentage; a negative value indicates a decrease, while a positive value indicates an increase relative to the reference value given by “Before”.

Figure 1 presents the proportion of participants who increased, did not change, or reduced their total leisure-time PA or MVPA volumes within each subgroup based on changes in self-perceived physical health, mental health, or sleep quality. Panels A and B show that 73% and 56% of participants who considered having better physical health had increased their total PA and MVPA volumes, while 60% and 49% of those who considered having worse physical health had decreased their total PA and MVPA volumes (*p* < 0.0001 for comparison between better vs. worse groups). During the lockdown, a lower proportion of participants not doing PA was observed among those who considered their physical health as better, while the proportion of inactive persons increased among those whose physical health was worse (*p* < 0.0001 in the zero-inflated model) (Table 3). Accordingly, total PA increased (~90 min/week) in participants who perceived their physical health as better and decreased (~64 min/week) in those who considered their physical health as worse (*p* < 0.0001). Similar results were observed for LPA and MVPA.

**Table 3:** Selected components of the 24 h movement behaviours before and during lockdown according to changes in self-perceived physical health.

Variables		Before Mean (95% CI)	During Mean (95% CI)	Before Mean (95% CI)	During Mean (95% CI)	Before Mean (95% CI)	During Mean (95% CI)	*p* Values		
								Group	Time	Group X Time
		**Changes in Self-Perceived Physical Health**								
		**Better (*n* = 264)**		**Unchanged (*n* = 1339)**		**Worse (*n* = 664)**				
Total PA *, min/week	Zero-inflated	18.9% (14.7%, 24.1%)	8.0% (5.2%, 11.9%)	18.5% (16.5%, 20.7%)	19.9% (17.8%, 22.1%)	15.7% (13.1%, 18.6%)	31.8% (28.3%, 35.4%)	0.0004	0.9600	<0.0001
		109.4 (97.2, 123.2)	196.6 (175.4, 220.4)	122.0 (115.4, 129.0)	120.6 (113.9, 127.6)	147.5 (136.6, 159.3)	83.8 (77.2, 91.1)	<0.0001	<0.0001	<0.0001
LPA *, min/week	Zero-inflated	38.3% (32.6%, 44.3%)	28.0% (22.9%, 33.8%)	34.0% (31.5%, 36.6%)	33.5% (31.1%, 36.1%)	35.8% (32.3%, 39.6%)	44.3% (40.5%, 48.1%)	0.0051	0.3892	<0.0001
		68.8 (59.8, 79.1)	118.4 (103.6, 135.3)	77.4 (72.1, 83.1)	82.1 (76.5, 88.1)	83.6 (76.1, 91.9)	59.7 (54.1, 65.9)	0.0027	<0.0001	<0.0001
MVPA *, min/week	Zero-inflated	42.4% (36.6%, 48.5%)	27.3% (22.2%, 33.0%)	45.9% (43.3%, 48.6%)	52.4% (49.7%, 55.0%)	36.9% (33.3%, 40.6%)	64.6% (60.9%, 68.2%)	<0.0001	<0.0001	<0.0001
		61.1 (52.8, 70.6)	96.6 (84.4, 110.5)	67.2 (61.3, 73.7)	63.8 (57.9, 70.3)	87.1 (78.7, 96.5)	51.7 (45.7, 58.5)	<0.0001	<0.0001	<0.0001
Screen time ^†^, min/day		140.4 (130.1, 151.5)	189.4 (175.5, 204.5)	142.3 (137.5, 147.1)	197.5 (190.9, 204.3)	135.2 (128.9, 141.9)	211.2 (201.3, 221.6)	0.7039	<0.0001	<0.0001
Sleep, hours/day		7.4 (7.2, 7.5)	7.5 (7.3, 7.6)	7.5 (7.4, 7.6)	7.3 (7.3, 7.4)	7.5 (7.4, 7.6)	7.1 (7.0, 7.2)	0.2521	<0.0001	<0.0001

CI, confidence interval; LPA, light-intensity physical activity; MVPA, moderate-to-vigorous-intensity physical activity; PA, physical activity. * Physical activity performed during leisure time; ^†^ Recreational screen time.

Regarding mental health, Figure 1C,D show that 46% and 26% of participants who self-perceived their mental health as better increased their total PA and MVPA, respectively, whereas 42% and 34% of those who considered their mental health as worse decreased their total PA and MVPA, respectively (*p* < 0.0001 for total PA and *p* = 0.0191 for MVPA for comparison between better vs. worse groups). The zero-inflated portion of the model indicated that the proportion of participants who did not perform PA decreased in those who self-perceived their mental health as better and increased in those who self-perceived their mental health as worse (*p* = 0.0055) (Table 4). This resulted in an increase in min/week of total PA among participants whose mental health improved and a decrease in those whose mental health deteriorated (*p* < 0.0001). Similar results were observed for LPA. Differences were also observed for sleep time, where it increased in the group that self-perceived their mental health as better and decreased in the group that self-perceived their mental health as worse (*p* < 0.0001).

**Table 4:** Selected components of the 24 h movement behaviours before and during lockdown according to changes in self-perceived mental health.

Variables		Before Mean (95% CI)	During Mean (95% CI)	Before Mean (95% CI)	During Mean (95% CI)	Before Mean (95% CI)	During Mean (95% CI)	*p* Values		
								Group	Time	Group X Time
		**Changes in Self-Perceived Mental Health**								
		**Better (*n* = 179)**		**Unchanged (*n* = 1099)**		**Worse (*n* = 987)**				
Total PA *, min/week	Zero-inflated	20.1% (14.9%, 26.6%)	17.3% (12.5%, 23.6%)	17.2% (15.1%, 19.6%)	20.1% (17.8%, 22.6%)	17.9% (15.7%, 20.5%)	25.0% (22.4%, 27.8%)	0.1986	0.0509	0.0055
		123.3 (106.3, 143.0)	132.2 (114.1, 153.0)	128.1 (120.4, 136.2)	125.5 (117.8, 133.6)	134.4 (126.0, 143.5)	110.6 (103.4, 118.4)	0.1589	<0.0001	<0.0001
LPA *, min/week	Zero-inflated	37.4% (30.6%, 44.8%)	31.8% (25.4%, 39.0%)	34.6% (31.8%, 37.4%)	34.9% (32.1%, 37.7%)	35.1% (32.1%, 38.1%)	38.3% (35.3%, 41.4%)	0.5486	0.5755	0.0375
		73.9 (62.4, 87.5)	85.2 (72.3, 100.4)	79.1 (73.1, 85.6)	84.4 (78.0, 91.3)	80.4 (74.2, 87.2)	75.1 (69.1, 81.5)	0.4160	0.0119	<0.0001
MVPA *, min/week	Zero-inflated	41.9% (34.9%, 49.3%)	46.9% (39.7%, 54.3%)	44.4% (41.5%, 47.4%)	51.8% (48.8%, 54.7%)	41.4% (38.4%, 44.6%)	55.6% (52.5%, 58.7%)	0.5246	<0.0001	0.0009
		75.1 (62.7, 90.0)	74.3 (61.6, 89.6)	76.3 (69.2, 84.0)	72.1 (65.0, 79.9)	80.1 (72.9, 88.0)	69.1 (62.2, 76.7)	0.3495	<0.0001	0.0181
Screen time ^†^, min/day		142.6 (130.0, 156.4)	191.0 (174.1, 209.6)	142.5 (137.3, 147.9)	193.1 (186.0, 200.4)	136.8 (131.5, 142.3)	211.0 (202.8, 219.5)	0.6109	<0.0001	<0.0001
Sleep, hours/day		7.4 (7.2, 7.6)	7.7 (7.5, 7.9)	7.4 (7.4, 7.5)	7.4 (7.3, 7.5)	7.6 (7.5, 7.7)	7.1 (7.0, 7.1)	0.0040	0.0400	<0.0001

CI, confidence interval; LPA, light-intensity physical activity; MVPA, moderate-to-vigorous-intensity physical activity; PA, physical activity. * Physical activity performed during leisure time; ^†^ Recreational screen time.

Figure 1E,F show that 59% and 37% of those who perceived their sleep quality as better had increased their total PA and MVPA, respectively. In comparison, 43% and 36% of those who perceived their sleep quality as worse decreased their total PA and MVPA, respectively (*p* < 0.0001 for comparison between better vs. worse groups). Table 5 shows essentially similar observations for total PA, LPA, and MVPA for the zero-inflated portion of the model and duration as for self-perceived physical health and mental health. Moreover, noticeable changes in sleep duration were observed, with an increase of 1 h/day in participants with better sleep quality and a decrease of 0.8 h/day among those with worse sleep quality (*p* < 0.0001). An increase in recreational screen time was observed in all groups. However, this increase was significantly greater among participants who reported a deterioration of their self-perceived physical and mental health as well as their sleep quality (*p* < 0.0001).

**Table 5:** Selected components of the 24 h movement behaviours before and during lockdown according to changes in self-perceived sleep quality.

Variables		Before Mean (95% CI)	During Mean (95% CI)	Before Mean (95% CI)	During Mean (95% CI)	Before Mean (95% CI)	During Mean (95% CI)	*p* Values		
								Group	Time	Group X Time
		**Changes in Self-Perceived Sleep Quality**								
		**Better (*n* = 185)**		**Unchanged (*n* = 1073)**		**Worse (*n* = 1004)**				
Total PA *, min/week	Zero-inflated	20.0% (14.8%, 26.4%)	14.1% (9.7%, 19.9%)	18.9% (16.7%, 21.4%)	21.4% (19.1%, 24.0%)	16.0% (13.9%, 18.4%)	24.1% (21.6%, 26.9%)	0.4847	0.2887	<0.0001
		118.1 (102.1, 136.6)	157.4 (136.6, 181.3)	130.2 (122.3, 138.6)	122.0 (114.5, 130.0)	132.9 (124.7, 141.6)	110.5 (103.4, 118.1)	0.1131	<0.0001	<0.0001
LPA *, min/week	Zero-inflated	40.5% (33.7%, 47.8%)	35.7% (29.1%, 42.8%)	35.8% (33.0%, 38.7%)	35.3% (32.5%, 38.2%)	33.3% (30.4%, 36.3%)	37.1% (34.1%, 40.1%)	0.6867	0.7145	0.0169
		71.0 (59.6, 84.5)	96.2 (81.2, 114.0)	79.0 (73.1, 85.5)	81.2 (75.1, 87.9)	81.3 (75.1, 88.0)	77.0 (71.0, 83.5)	0.1101	0.0577	<0.0001
MVPA *, min/week	Zero-inflated	42.2% (35.3%, 49.4%)	37.8% (31.1%, 45.0%)	45.1% (42.2%, 48.1%)	52.5% (49.5%, 55.5%)	40.5% (37.5%, 43.6%)	56.4% (53.3%, 59.4%)	0.0423	<0.0001	<0.0001
		73.3 (61.4, 87.4)	86.4 (72.8, 102.7)	79.4 (71.9, 87.6)	73.6 (66.3, 81.7)	77.0 (70.2, 84.5)	65.2 (58.7, 72.3)	0.4224	<0.0001	<0.0001
Screen time ^†^, min/day		135.1 (123.3, 148.0)	191.2 (174.6, 209.4)	143.6 (138.2, 149.1)	195.8 (188.5, 203.3)	136.9 (131.7, 142.4)	207.3 (199.3, 215.5)	0.6010	<0.0001	<0.0001
Sleep, hours/day		7.2 (7.0, 7.3)	8.2 (8.0, 8.4)	7.5 (7.4, 7.5)	7.6 (7.5, 7.7)	7.6 (7.5, 7.7)	6.8 (6.8, 6.9)	<0.0001	0.0009	<0.0001

CI, confidence interval; LPA, light-intensity physical activity; MVPA, moderate-to-vigorous-intensity physical activity; PA, physical activity. * Physical activity performed during leisure time; ^†^ Recreational screen time.

To examine changes in leisure-time PA level during the COVID-19 lockdown, participants were categorized according to their weekly PA volume before the lockdown (Figure 2). Participants who were the most active (≥300 min/week) before the lockdown decreased total PA (95% CI: −131.7, −81.6; *p* < 0.0001), LPA (95% CI: −53.1, −15.3; *p* = 0.0004), and MVPA (95% CI: −87.4, −57.5; *p* < 0.0001) while those who were inactive increased their total PA (95% CI: 29.7, 51.6; *p* < 0.0001), LPA (95% CI: 18.1, 35.9, *p* < 0.0001), and MVPA (95% CI: 8.0, 19.2, *p* < 0.0001).

Table 6 presents the odds of considering physical health, mental health, and sleep quality as better according to an increase in selected components of the 24 h movement behaviours after adjusting for age, BMI, baseline total PA (model 1) or baseline LPA and MVPA (model 2), diabetes, hypertension, hypercholesterolemia, heart disease, administrative regions, education, household income, and working status. Participants increasing weekly MVPA by 30 min were 1.65 times more likely to self-perceive physical health as better (95% CI: 1.51, 1.81; *p* < 0.0001). Furthermore, participants who added 1 h of sleep per day had 1.51 greater odds of considering their mental health as better (95% CI: 1.32, 1.73; *p* < 0.0001). Adding 1 h of sleep per day was also associated with higher odds of considering sleep quality as better (OR: 2.84, 95% CI: 2.37, 3.40; *p* < 0.0001). Finally, an increase of 30 min per day in recreational screen time was associated with lower odds of considering physical health (OR: 0.93, 95% CI: 0.88, 0.99; *p* = 0.0200), mental health (OR: 0.90, 95% CI: 0.85, 0.96; *p* = 0.0004), and sleep quality (OR: 0.94, 95% CI: 0.88, 1.00; *p* = 0.0432) as better.

**Table 6:** Odds ratio for reporting improved self-perceived physical health, mental health, and sleep quality during the lockdown based on changes in physical activity, screen time, and sleep time.

Variables	Univariable			Multivariable					
				Model 1			Model 2		
	OR	95% CI	*p* Value	OR	95% CI	*p* Value	OR	95% CI	*p* Value
Better self-perceived physical health (*n* = 865–928)									
Total PA *, 30 min increase/week	1.36	(1.30, 1.43)	<0.0001	1.40	(1.32, 1.47)	<0.0001			
LPA *, 30 min increase/week	1.24	(1.18, 1.31)	<0.0001				1.27	(1.19, 1.35)	<0.0001
MVPA *, 30 min increase/week	1.64	(1.50, 1.79)	<0.0001				1.65	(1.51, 1.81)	<0.0001
Screen time ^†^, 30 min increase/day	0.92	(0.89, 0.96)	0.0002	0.93	(0.87, 0.98)	0.0094	0.93	(0.88, 0.99)	0.0200
Sleep time, 1 h increase/day	1.22	(1.10, 1.36)	0.0001	1.27	(1.11, 1.45)	0.0004	1.27	(1.11, 1.46)	0.0005
Gender, women	1.34	(0.90, 2.01)	0.1556	0.94	(0.56, 1.58)	0.8198	0.98	(0.57, 1.65)	0.9243
Better self-perceived mental health (*n* = 1082–1166)									
Total PA *, 30 min increase/week	1.06	(1.03, 1.09)	<0.0001	1.07	(1.03, 1.10)	<0.0001			
LPA *, 30 min increase/week	1.06	(1.02, 1.10)	0.0034				1.06	(1.02, 1.11)	0.0044
MVPA *, 30 min increase/week	1.08	(1.03, 1.14)	0.0017				1.08	(1.02, 1.14)	0.0145
Screen time ^†^, 30 min increase/day	0.91	(0.87, 0.96)	0.0002	0.90	(0.85, 0.96)	0.0004	0.90	(0.85, 0.96)	0.0004
Sleep time, 1 h increase/day	1.46	(1.30, 1.65)	<0.0001	1.51	(1.32, 1.73)	<0.0001	1.51	(1.32, 1.73)	<0.0001
Gender, women	0.81	(0.53, 1.24)	0.3288	0.79	(0.49, 1.29)	0.3457	0.79	(0.49, 1.29)	0.3465
Better self-perceived sleep quality (*n* = 1101–1189)									
Total PA *, 30 min increase/week	1.11	(1.08, 1.14)	<0.0001	1.13	(1.09, 1.17)	<0.0001			
LPA *, 30 min increase/week	1.10	(1.06, 1.14)	<0.0001				1.12	(1.07, 1.18)	<0.0001
MVPA *, 30 min increase/week	1.15	(1.09, 1.21)	<0.0001				1.13	(1.06, 1.19)	<0.0001
Screen time ^†^, 30 min increase/day	0.96	(0.92, 1.01)	0.0802	0.94	(0.88, 1.00)	0.0421	0.94	(0.88, 1.00)	0.0432
Sleep time, 1 h increase/day	2.61	(2.25, 3.04)	<0.0001	2.84	(2.37, 3.40)	<0.0001	2.84	(2.37, 3.40)	<0.0001
Gender, women	0.75	(0.49, 1.14)	0.1776	0.49	(0.29, 0.83)	0.0084	0.49	(0.29, 0.83)	0.0084

CI, confidence interval; LPA, light-intensity physical activity; MVPA, moderate-to-vigorous-intensity physical activity; OR, odds ratio; PA, physical activity. * Physical activity performed during leisure time; ^†^ Recreational screen time. Models were adjusted for age, body mass index, baseline total PA (model 1) or baseline LPA and MVPA (model 2), diabetes, hypertension, hypercholesterolemia, heart disease, administrative regions, education, household income, and working status.

As exploratory analyses, we were also interested in examining the potential contribution of gender and the type of work (healthcare vs. non-healthcare workers) to the differences observed (Supplementary Figures S2–S4 and Supplementary Table S1). For the selected components of the 24 h movement behaviours, both men and women increased recreational screen time to the same extent, but men decreased total leisure-time PA (*p* = 0.0002) and MVPA (*p* < 0.0001) to a greater extent than women (Supplementary Figure S2). When men and women were classified according to weekly leisure-time PA volume before the lockdown (Supplementary Figure S3), only men doing 150–299 min/week of total PA decreased to a greater extent than women (*p =* 0.0009). Moreover, when compared according to MVPA volume before the lockdown (Supplementary Figure S4), the greatest difference was observed for men and women in the subgroup doing ≥150 min/week (*p =* 0.0036).

Finally, we were interested in documenting selected components of the 24 h movement behaviours before and during the lockdown in healthcare vs. non-healthcare workers (Supplementary Table S1). Recreational screen time increased during the lockdown to a greater extent among non-healthcare workers (*p* < 0.0001). The zero-inflated portion of the statistical model revealed that the increase in the proportion of participants not doing leisure-time PA was similar between healthcare and non-healthcare workers (*p =* 0.1477). However, healthcare workers decreased their total PA time to a greater extent than non-healthcare workers (*p* < 0.0001). Moreover, there was a greater increase in the proportion of individuals not doing MVPA among healthcare workers than non-healthcare workers in the zero-inflated portion of the model (*p* = 0.0038), as well as a greater decrease in MVPA duration (*p* = 0.0367). Finally, the proportion reaching PA guidelines decreased significantly more in healthcare workers (before: 23.8% [95% CI: 21.0, 26.9]; during: 17.3% [95% CI: 14.8, 20.1]) than non-healthcare (before: 15.7% [95% CI: 13.5, 18.1]; during: 15.0% [95% CI: 12.8, 17.4]) workers (*p* = 0.0085).

## 4. Discussion

The COVID-19 lockdown created a unique context to document the relationships between changes in PA and self-perceived physical health, mental health, and sleep quality among adults living in the province of Québec under constrained conditions.

### 4.1. Heterogeneity in Physical Activity Levels in Response to the Lockdown

Total leisure-time PA expressed in minutes/week globally decreased by 9.1% during the lockdown, corresponding to a reduction of 9.5% in MVPA. Given that baseline PA levels were already well below the Canadian 24 h movement guidelines prior to COVID-19, this subsequent decline represents a critical shift further away from public health targets, widening an already significant physical inactivity gap. The decrease in total PA and MVPA observed in the present study is in line with findings previously reported during the first wave of the COVID-19 pandemic elsewhere around the world [5,6,7,9,24]. Moreover, in a systematic review paper of Nindenshuti and Caire-Juvera [25], a decrease in PA level was reported in all of the 25 studies listed. In a meta-analysis of 173 studies [26], a reduction in total PA was reported, the decrease being observed for all intensity levels (light, moderate or vigorous). Most importantly and consistent with our findings, moderator analyses showed no influence of sex, BMI, health status, pre-activity level and assessment method (objective and subjective) on PA. However, results of a meta-analysis by Wunsch et al. [10] revealed a slight negative, but non-significant trend towards a decrease in PA due to the COVID-19 pandemic. This paper also documented that most self-reported and all device-based measurement methods consistently showed a reduction in PA.

In the present study, we found individual heterogeneity in behavioural responses to the lockdown. For instance, people who were either inactive or the least active before the lockdown increased total PA, LPA, and MVPA, whereas people who were the most active before the lockdown decreased their PA levels (total, LPA, and MVPA). Despite the fact that we cannot exclude the possibility that these observations may be, at least partly, due to the regression to the mean effect, these findings are nevertheless concordant with other studies reporting a substantial decrease in PA among previously active individuals [5,7,9,27]. This could indicate that very active individuals may have had greater difficulties finding other PA options while inactive people may have taken advantage of more time being suddenly available to introduce simple PA behaviours such as walking. For most highly active individuals, practicing a large volume of PA often required the use of sports infrastructure located outside a 1 km perimeter of one’s home, which corresponded to the travel distance limit in Québec at the time of the pandemic. However, an online survey conducted in Canada on a sample mostly living in western Canada has shown that previously active individuals were less likely to decrease their PA than inactive individuals, indicating possible regional differences in Canada, as each province had its own restrictions put in place [28]. Finally, men decreased total leisure-time PA and MVPA to a greater extent than women. The decrease in MVPA was mainly observed in individuals doing more than 150 min of MVPA per week, men showing a greater reduction compared to women. This finding was essentially similar to another study conducted in Spain that reported that men reduced their moderate activities while women increased moderate activities during lockdown [5].

### 4.2. Insufficient Physical Activity: Implication for Population Health

Before the lockdown, only 19% of participants reached the Canadian 24 h movement guidelines of 150 min/week of MVPA, a proportion that even further decreased during the lockdown, a figure which is clearly a source of concern from a population health standpoint [23]. Furthermore, among healthcare workers, only 24% reached PA guidelines before the lockdown, with this number dropping to 17% during the lockdown. This decline should also be a source of concern for our healthcare authorities, especially considering the tremendous pressure under which our healthcare workers were at the time and still are post-pandemic. The decrease in adults reaching PA guidelines in our study is not an isolated finding. A study conducted by Statistics Canada has shown that, in the province of Québec, the proportion of adults (18–64 years) reaching MVPA guidelines decreased from 59.1% to 52.5% between early 2020 and the end of 2020 [29]. The substantial difference between these proportions (~50–60% vs. ~15–20%) probably resides in the fact that reports from Statistics Canada consider all types of MVPA (recreation, transportation, and household and/or occupational), while the present analyses focused solely on leisure-time aerobic MVPA. In Canada, the percentage of Canadian adults reaching the PA recommendations has remained relatively stable between early 2020 and the end of 2020 [29,30].

### 4.3. Leisure-Time Physical Activity and Self-Perceived Physical Health

Another relevant finding in our study was the link between leisure-time PA and self-perceived physical health. For instance, participants who considered themselves in better physical health during the lockdown had increased their volume of total PA, LPA, and MVPA, while those who considered themselves in worse physical health had decreased all levels of leisure-time PA. In our study, 73% of the participants who perceived their physical health as better increased their total PA level and 56% increased their MVPA, supporting the association between self-perceived health and PA. Furthermore, adding 30 min of MVPA per week was strongly associated with physical health; participants had 65% higher odds of reporting better self-perceived physical health. This finding was also observed in a study by AlDukhail and Bahdila [31] published in 2022, in which they observed an increased risk of poor self-perception of health among inactive individuals before and during the COVID-19 pandemic. Of course, these results are concordant with many studies linking PA and self-perceived health in various age groups [32,33]. Our study adds to this evidence by showing that changes in PA practice during the COVID-19 pandemic were also linked to changes in self-perceived physical health.

### 4.4. Leisure-Time Physical Activity and Self-Perceived Mental Health

In addition to physical health, leisure-time PA was also related to self-perceived mental health during the lockdown. For instance, the 179 participants who considered their mental health as better had increased their total PA and LPA compared to a decrease in all PA variables among the 987 participants who considered their mental health as worse. However, as only LPA increased in the “better” mental health group, the link between PA and health perception appears to be stronger for physical health than mental health. These results are in line with other studies that have shown that a decrease in PA during the pandemic was associated with a greater risk of depression, loneliness, stress/anxiety, and worse perception of mental health [8,34,35]. It is also important, however, to keep in mind that the present study does not provide evidence regarding the direction of the PA—mental health relationship. For instance, as reviewed by Dubash [36], there may be a lagged prospective effect of depressive symptoms on PA, although a considerable body of literature has also documented the rather rapid effects of PA on mental health [36,37]. Although our study cannot address this issue, it nevertheless provides further evidence of a dynamic link between PA and mental health.

Recreational screen time, which can be used to estimate sedentary behaviour, also increased (by more than 43%), exceeding Canadian 24 h movement guidelines which recommend a maximum of 180 min/day of recreational screen time (outside of work) [23]. This increase in sedentary behaviour has been reported in many studies around the world [3,4,5,24]. A systematic review paper reported a universal increase in sedentary behaviour in 13 out of 13 studies [25]. A comprehensive meta-analysis of 173 studies found a significant increase in sedentary behaviour during periods of public health restrictions [26]. As sedentary behaviour and PA are proven to be associated with cardiovascular and cardiometabolic diseases as well as mental health (especially depression) [38,39,40], the lockdown in the province of Québec may have important implications for population health.

### 4.5. Sleep

It is now well recognized that sleep plays an important role in general and cardiometabolic health [41]. As a result, the American Heart Association’s Life’s Essential 8 recommendations to optimize cardiovascular health now consider regular sleep schedule and sleep quality as relevant behavioural targets [42]. Therefore, it was of interest to explore the association between sleep quality and time, leisure-time PA, and health during the lockdown. Overall, sleep time decreased (~3%) during the lockdown, and 44% of the sample reported a decrease in sleep quality. Although of small magnitude, a chronic reduction in sleep duration at the population level may have serious health consequences for physical and mental health [43]. Other studies conducted during the pandemic have also shown a decrease in sleep quality and an increase in psychological symptoms, with the exception of people with extreme insomnia [9,15,16,17,44,45]. In our study, those who perceived their sleep quality as better had increased total PA, LPA, and MVPA volumes as well as their sleep time. However, participants who perceived their sleep quality as worse decreased total PA, LPA, and MVPA, as well as sleep time. These results are consistent with studies linking PA and better sleep quality in various age groups [46,47,48]. In addition to the association between leisure-time PA and self-perceived sleep quality, our study shows that sleep time is also an important factor to consider when looking at physical and mental health. Indeed, sleep time decreased in participants who considered their physical health as worse. Sleep time also decreased in the “worse” mental health group while it increased in the “better” group. Furthermore, adding 1 h of sleep time per day was an important health variable relevant to mental health, as it increased by 51% the odds of reporting mental health as better. It is interesting to note that female gender turned out to be an important independent factor for self-perceived sleep quality after sleep duration, a result concordant with another Canadian study in which women reported lower sleep quality and efficiency than men [49].

### 4.6. Physical Activity, Screen Time and Sleep

The lifestyle triad of PA, recreational screen time, and sleep duration must also be simultaneously considered and addressed from a public health perspective. Indeed, while only 10.4% of our participants simultaneously met the guideline criteria for MVPA, screen time and age-specific sleep recommendations, this low frequency was even further reduced to 5.1% during the lockdown. From a population health perspective, public health policies and programmes as well as educational activities targeting health professionals and the lay public should be developed in order to put a greater focus on the importance of these critical behavioural drivers of physical and mental health.

### 4.7. Strengths and Limitations

The major strength of MAVIPAN is the scope of variables considered in this study. Indeed, MAVIPAN was designed not only to assess changes in healthy habits in the context of the lockdown, but also in mental and physical health as well as sleep quality. Another strength is the use of online questionnaires, which allowed individuals across Québec with Internet access to take part in the study, allowing participation from all administrative regions. However, despite providing useful insights into how healthy behaviours evolved in the context of the drastic measures implemented during the COVID-19 lockdown, our study has limitations. First, the use of a non-probabilistic purposive sampling approach is generally associated with biases which may lead to under- or over-representation of certain groups. For instance, our sample consisted predominantly of highly educated women mainly from two administrative regions. These characteristics limit the external validity and generalisability of our findings to the Québec population. Thus, prevalence estimates should be interpreted with caution, and these sampling characteristics should be considered when interpreting the observed associations between changes in PA and self-perceived health outcomes. This limitation was already acknowledged when the MAVIPAN protocol was published [20]. Another limitation is the lack of pre-pandemic measures. In the online survey, participants were asked to recall their leisure-time PA habits and self-perceived physical health, mental health, and sleep quality before the lockdown. Therefore, changes in these outcomes were retrospectively self-reported, which may have introduced recall bias as well as social desirability bias, whereby participants may have modified their responses to align with perceived societal expectations or to present themselves in a favourable light. In addition, the reliance on self-reported measures for both exposures (e.g., PA, screen time, sleep) and outcomes (self-perceived health and sleep quality) may have introduced a common-method bias, potentially inflating the observed associations. We also acknowledge the use of non-validated questionnaires to assess PA (rather than objective measures), as well as screen time and sleep variables, which may have introduced measurement error and misclassification. Moreover, given the cross-sectional nature of the present analyses, reverse causation cannot be ruled out, as individuals with better self-perceived sleep quality and health may have been more likely to engage in higher levels of PA. In addition, the lack of adjustment for baseline physical and mental health should be considered, as these data were not collected. Finally, the grouping of self-perceived health responses may have reduced variability and masked subtle differences between adjacent categories.

## 5. Conclusions

The COVID-19 lockdown in the province of Québec during the first wave in the spring of 2020 was accompanied by changes in PA behaviours, with an overall decrease in total leisure-time PA driven by a reduction in MVPA. Beyond this specific context, our findings highlight the importance of total PA, including light-intensity activity, in relation to physical and mental health as well as sleep quality. These findings suggest that public health strategies may benefit from considering a broader range of movement behaviours, including recreational screen time and sleep, rather than focusing solely on MVPA, both in constrained and everyday conditions.

## Data Availability

The data generated and analyzed in this study are not publicly available for ethical reasons but are available from the corresponding author upon reasonable request and approval by the Institutional Review Board.

## Supplementary Materials

The following supporting information can be downloaded at: https://www.mdpi.com/article/10.3390/epidemiologia7040117/s1, Figure S1: Flowchart of participants and reasons for exclusion for the present analyses; Figure S2: Changes in minutes/day of recreational screen time and minutes/week of total leisure-time PA, LPA, and MVPA in men and women; Figure S3: Changes in total leisure-time PA in men and women according to total leisure-time PA practice before the lockdown; Figure S4: Changes in MVPA in men and women according to MVPA practice before the lockdown. Table S1: Selected components of the 24 h movement behaviours before and during lockdown in healthcare and non-healthcare workers at the time of the lockdown.

## Abbreviations

The following abbreviations are used in this manuscript:

BMI	Body mass index
CI	Confidence interval
CIUSSS	Centre intégré universitaire de santé et de services sociaux
LPA	Light-intensity physical activity
MAVIPAN	Ma vie et la pandémie
MVPA	Moderate-to-vigorous-intensity physical activity
OR	Odds ratio
PA	Physical activity
SARS-CoV-2	Severe acute respiratory syndrome coronavirus-2
