# Supplementary material for: Leisure-Time Physical Activity, Sleep, and Self-Perception of Health During the COVID-19 Lockdown in Québec
Source: Epidemiologia (Basel). 2026 Aug 20;7(4):117. doi: 10.3390/epidemiologia7040117 (PMC13512169; doi:10.3390/epidemiologia7040117)
Supplement: Supplementary file 1 [file epidemiologia-07-00117-s001.zip › epidemiologia-4409729-supplementary.pdf]

epidemiologia

Article

Leisure-time Physical Activity, Sleep, and Self-perception of Health during the COVID-19 Lockdown in Québec

Adrien Murphy-Després, Natalie Alméras, Dominic J. Chartrand, Isabelle Lemieux, Serge Simard, Laura Jalbert, Annie LeBlanc and Jean-Pierre Després

SUPPLEMENTARY MATERIAL – EXPLORATORY ANALYSES

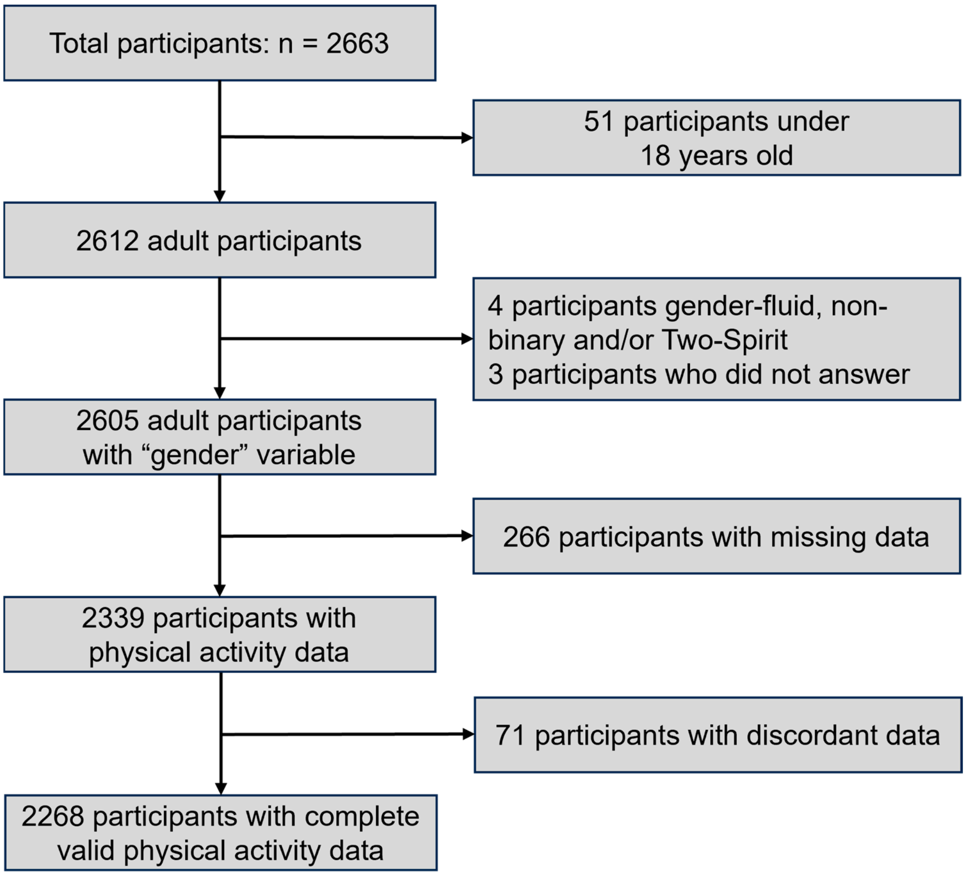

Figure S1: Flowchart of participants and reasons for exclusion for the present analyses.

 https://doi.org/10.3390/epidemiologia7040117

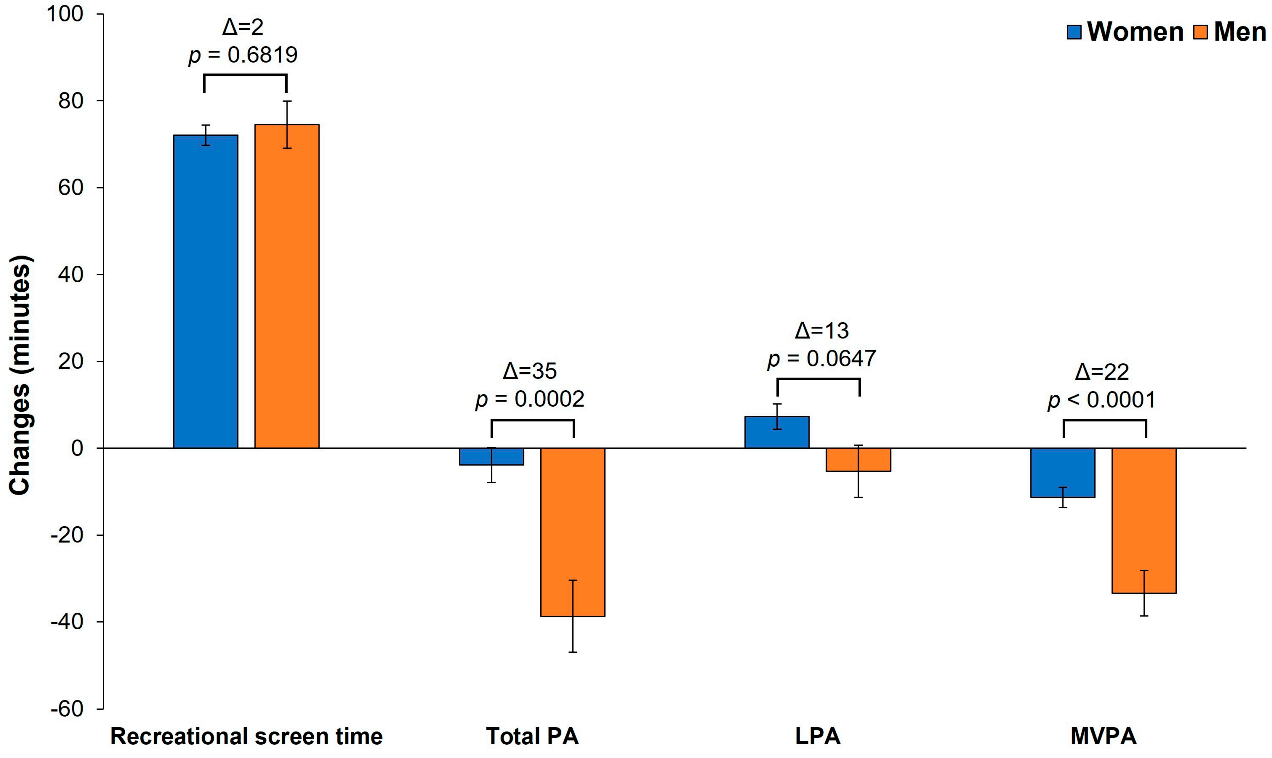

Figure S2. Changes in minutes/day of recreational screen time and minutes/week of total leisure-time physical activity (PA), light-intensity PA (LPA), and moderate-to-vigorous-intensity PA (MVPA) in men and women.

https://doi.org/10.3390/epidemiologia7040117

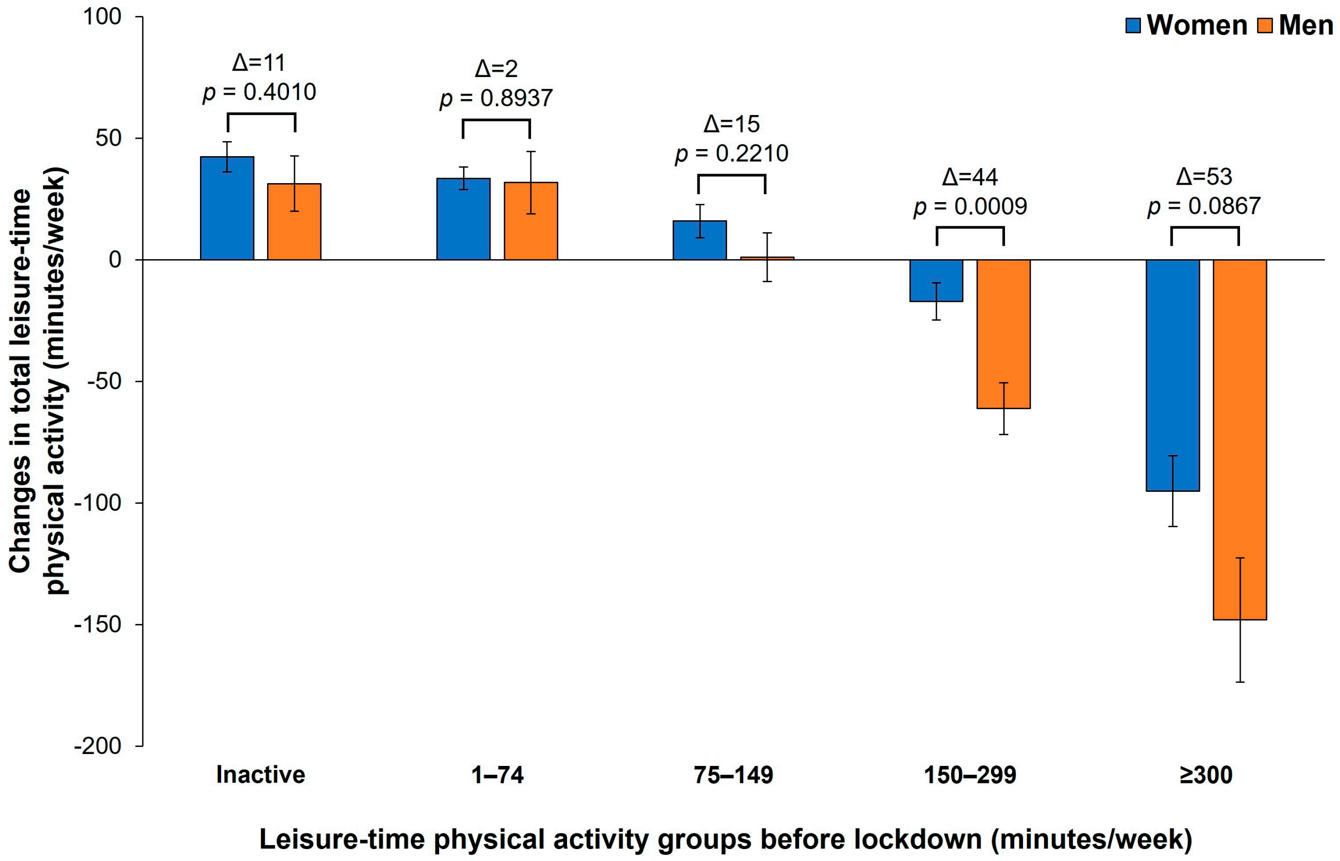

Figure S3. Changes in total leisure-time physical activity (PA) in men and women according to total leisure-time PA practice before the lockdown.

https://doi.org/10.3390/epidemiologia7040117

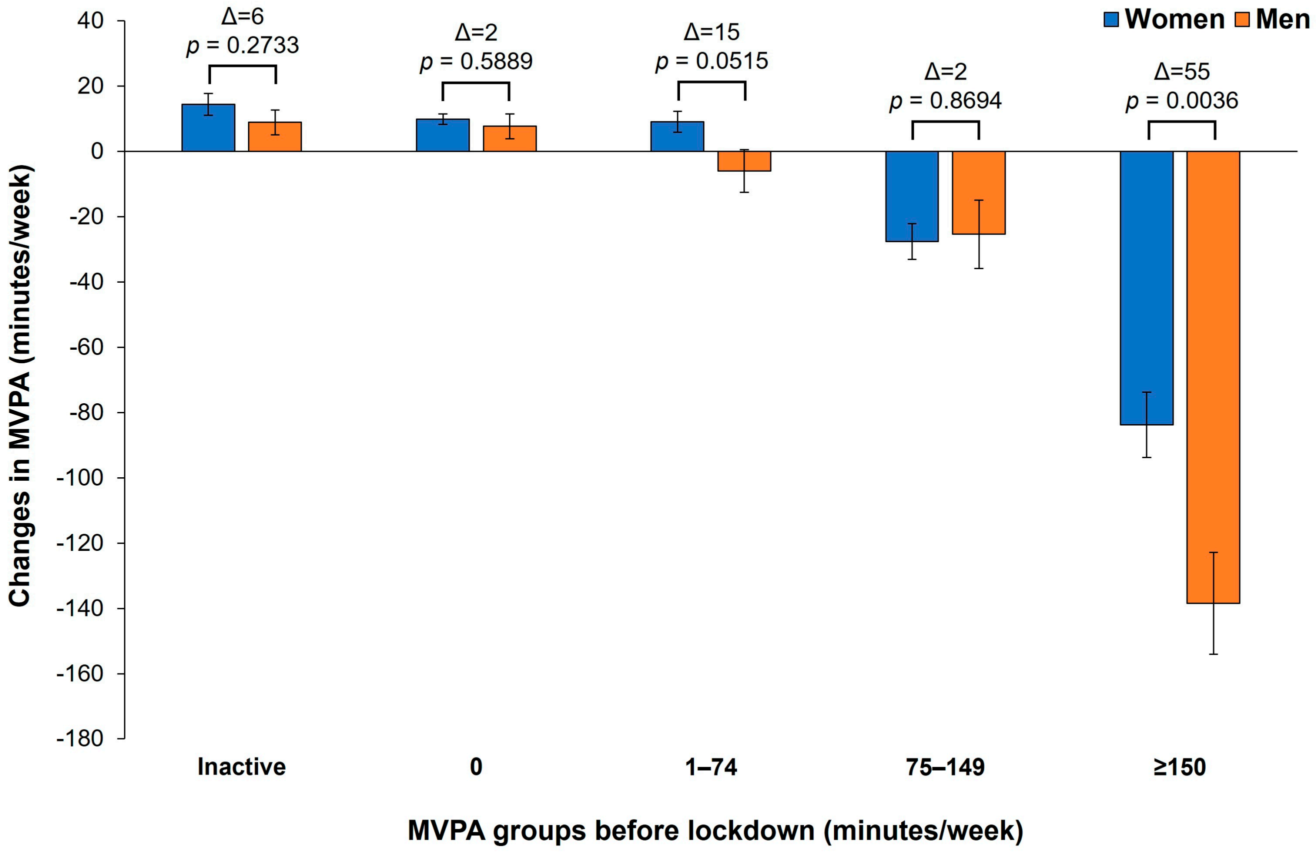

Figure S4. **Changes in moderate-to-vigorous-intensity physical activity (MVPA) in men and women according to MVPA practice before the lockdown.**

Table S1. Selected components of the 24-hour movement behaviours before and during lockdown in healthcare and non-healthcare workers at the time of the lockdown.

Variables		Healthcare Workers (*n* = 785)		Non-Healthcare Workers (*n* = 950)		*p* Values		
		Before Mean (95% CI)	During Mean (95% CI)	Before Mean (95% CI)	During Mean (95% CI)	Group	Time	Group X Time
Total PA *, min/week	Zero-inflated	14.1% (11.9%, 16.8%)	19.1% (16.5%, 22.0%)	18.5% (16.2%, 21.1%)	21.3% (18.8%, 24.0%)	0.0355	<0.0001	0.1477
		134.3 (125.3, 143.8)	114.6 (106.7, 123.0)	125.1 (117.2, 133.6)	122.9 (115.0, 131.4)	0.1028	0.7175	<0.0001
LPA *, min/week	Zero-inflated	35.9% (32.6%, 39.4%)	35.8% (32.5%, 39.2%)	36.2% (33.2%, 39.3%)	35.3% (32.3%, 38.4%)	0.9514	0.6218	0.7065
		73.4 (67.2, 80.1)	72.6 (66.5, 79.3)	78.8 (72.5, 85.7)	82.4 (75.8, 89.5)	0.1565	0.1621	0.2957
MVPA *, min/week	Zero-inflated	34.5% (31.3%, 37.9%)	48.3% (44.8%, 51.8%)	44.4% (41.3%, 47.6%)	52.1% (48.9%, 55.3%)	0.0011	<0.0001	0.0038
		87.5 (79.8, 95.9)	75.9 (68.5, 84.1)	75.3 (68.1, 83.3)	72.1 (64.7, 80.3)	0.0005	0.2704	0.0367
Screen time †, min/day		125.1 (119.9, 130.7)	166.5 (159.4, 173.8)	131.6 (126.6, 136.9)	198.1 (190.5, 206.0)	<0.0001	<0.0001	<0.0001
Sleep, hours/day		7.6 (7.5, 7.7)	7.3 (7.2, 7.4)	7.4 (7.4, 7.5)	7.3 (7.3, 7.4)	0.2730	<0.0001	0.0034

CI, confidence interval; LPA, light-intensity physical activity; MVPA, moderate-to-vigorous-intensity physical activity; PA, physical activity. * Physical activity performed during leisure time; † Recreational screen time.

https://doi.org/10.3390/epidemiologia7040117
